# Low Molecular Weight Hyaluronic Acid Added to Six Specific Amino Acids in the Treatment of Striae Alba (SA): An Observational Study

**DOI:** 10.1007/s00266-024-03911-8

**Published:** 2024-04-01

**Authors:** Elena Fasola, Vincenzo Nobile

**Affiliations:** 1 Microsurgeon at Gyplast Medical Institute, 20129 Milan, MI Italy; 2 R&D Department, Complife Italia, 27028 San Martino Siccomario, PV Italy

**Keywords:** Striae distensae, Hyaluronic acid, Amino acids, Intra-mural injection

## Abstract

**Abstract:**

Striae distensae or stretch marks are a common complaint among women and can be distressing. The present study aimed to assess the efficacy of a mixture of low molecular weight hyaluronic acid and six amino acids when applied with a specific intradermal injection technique known as intra-mural fluid technique. A clinical study was carried out in 32 patients (with a dropout rate by 9.4%) with striae distensae alba (SA) in one or more of the following anatomical areas: breast, abdomen, inner thigh, trochanteric area, gluteal area, posterior supra-iliac area, and lumbar area. Product efficacy was assessed by the investigator using the Global Aesthetic Improvement Scale, while a Likert scale was used to evaluate to score the treatment tolerability and a QoL stretch marks questionnaire was used to investigate the patients’ self-body image. The treatment was effective in improving the appearance of SA fifteen days after the second treatment and 6 months after the first treatment (and after a total of 4 treatments). The product efficacy and tolerability were also perceived by the patients during each treatment session. Our results suggest that the test treatment is a valid treatment option to decrease the appearance of SA.

**Level of Evidence IV:**

This journal requires that authors assign a level of evidence to each article. For a full description of these Evidence-Based Medicine ratings, please refer to the Table of Contents or the online Instructions to Authors https://www.springer.com/00266

## Introduction

Striae distensae (SD) or stretch marks are linear scars of the skin, mainly localized on the gluteal-trochanteric area, abdomen, hips, thighs, and breasts. The prevalence of SD is up to 90% of the general population being more common in females than in males [1, 2]. SD generally appear during puberty, pregnancy (Striae Gravidarum, SG), and in case of rapid variation in body weight [3]. Even if the pathogenetic mechanism remain unclear, the main hypothesis is structural change of dermal collagen, fibrillin, fibronectin and elastin [4, 5]. The risk factors of SD are well known and include genetic, hormonal changes and mechanical stress [6]. The occurrence of SD is also associated with diseases (e.g., Cushing’s or Marfan syndrome) [7–10], with systemic corticosteroids or other drugs therapy (including chemotherapy, contraceptives, and prolonged antibiotic therapy) [11], and with plastic surgery [12, 13]. Even if SD are not a life-threatening condition, it is often cause of cosmetic morbidity and psychological distress, especially during the adolescence and in professions where the physical appearance is of high importance [11, 14, 15].

The first stage of SD is characterized by smooth, irritable, raised, and erythematous early lesions, and is known as striae rubra (SR); while at a latter stage, when aging of the lesion occurs, they appear pale, flat, and irregular and are known as striae alba (SA) [11]. Histologically SA show an excess of thin elastic fibers in the papillary dermis area and thicker fibers in the periphery associated with vasodilation, edema, and a decrease of elastin and fibrillin fibers [1]. On the other hand, the histological examination of SA reveals epidermal atrophy, decreased vascularization, and more thin, dense, and atrophic collagen lines [11, 16, 17]. Several studies reported a key role of fibroblasts in the pathogenesis of striae. Compared with normal fibroblasts, expression of fibronectin and both type I and III procollagen and collagen type IV were found to be significantly reduced in fibroblasts from striae, suggesting that there exist fundamental aberrations of fibroblast metabolism in SD [18].

The onset of SD is characterized by an overproduction of collagen and elastin fiber and their alignment. Histologically specimens shows densely packed elastin and collagen fibers aligned parallel to the skin, in contrast to the random orientation of the fibers in healthy skin [19]. Using electronic microscopy, Sheu et al. visualized elastolysis in the dermis accompanied by mast cells degranulation and influx of activate macrophages enveloping fragmented elastic fibers [20], while a distortion of dermal papillae was reported using the reflectance confocal microscopy by Rolfe et al. [21]. An active involvement of dermal collagen in SD onset, using confocal Raman microscopy, was reported by Lung et al [22].

Despite a plethora of therapies have been proposed for SD [23], their treatment is a great challenge for professionals since the results obtained are not always satisfactory and may have side effects. It is then desirable to investigate on new therapies to treat SD in an effective way and with a good safety profile. In this direction, injectable dermal fillers, as monotherapy or combination therapy, have been used as a safe treatment for SD with minimal adverse effects [24]. Injection of hyaluronic acid (HA) has become a worldwide standard procedure to provide soft tissue augmentation and correct soft tissue defects [25]. The putative mechanism of action for HA efficacy, beyond its viscoelastic and filling properties, is related to the enhancement of the synthesis of the components in the extracellular matrix (ECM) [26, 27]. Treatment of stretch marks by HA injection has been demonstrated to be effective in the improvement of SA appearance. The injection of HA by a high-velocity pneumatic injector showed an improvement of SA after 2/3 months of treatment [28–30]. In a previous preliminary study, the injection by a specific intradermal injection technique known as intra-mural fluid technique (ImFT) of a mixture of a low molecular weight hyaluronic acid and six amino acids (Sunekos Body®, Professional Dietetics, Milan, Italy) was reported to be effective in improving SD appearance after 2 injections at intervals of 2 weeks each [31]. The present study aimed to assess in a larger population, the efficacy of the test product, in decreasing the stretch marks appearance on subjects with SA in different body areas.

## Materials and Methods

### Study Design

This study was a prospective clinical study carried at one clinical center (Gyplast Medical Institute, Milan, Italy) between February 2022 and April 2023. It consisted of a screening/basal visit (T0), four treatment visits within 15 days of each other (T1–T4), and a follow-up visit after 6 months from the first treatment and after 4 treatments (T5).

All the study procedures were carried out according to the World Medical Association’s (WMA) Helsinki Declaration and its amendments. A signed informed consent form and the consent release form for the publication of photographs were obtained from all the subjects participating in the study before any study-related procedure took place.

Ethical review and approval were waived for this study due to observational nature of the study (the product was on the market before the study start). The ImFT is a minimally invasive technique used in the medical practice without any relevant side effect. Furthermore, the study treated only patients searching for a stretch marks treatment.

### Eligibility Criteria for Participants

The study enrolled all the female subjects requesting stretch marks treatment. Eligible participants were healthy adult female Caucasian subjects with BMI > 32 Kg/m^2^ and SA in one or more of the following anatomical areas: breasts, abdomen, inner thigh, trochanteric area, gluteal area, posterior supra-iliac area, and lumbar area. The study excluded patients with auto-immune soft tissues disease, with ongoing anti-inflammatory therapy, inflammatory or infection diseases affecting the treatment area, subjects with presumed or confirmed sensitivity toward one or more ingredients in the product formula, subjects with alimentary disorders and frequent weight variation. The study further excluded subjects planning to have any cosmetic, esthetic, or surgical procedure in the tested area. Any use of topical products that can interfere with the stretch marks appearance was prohibited.

### Description of Treatment

**Fig. 1:**
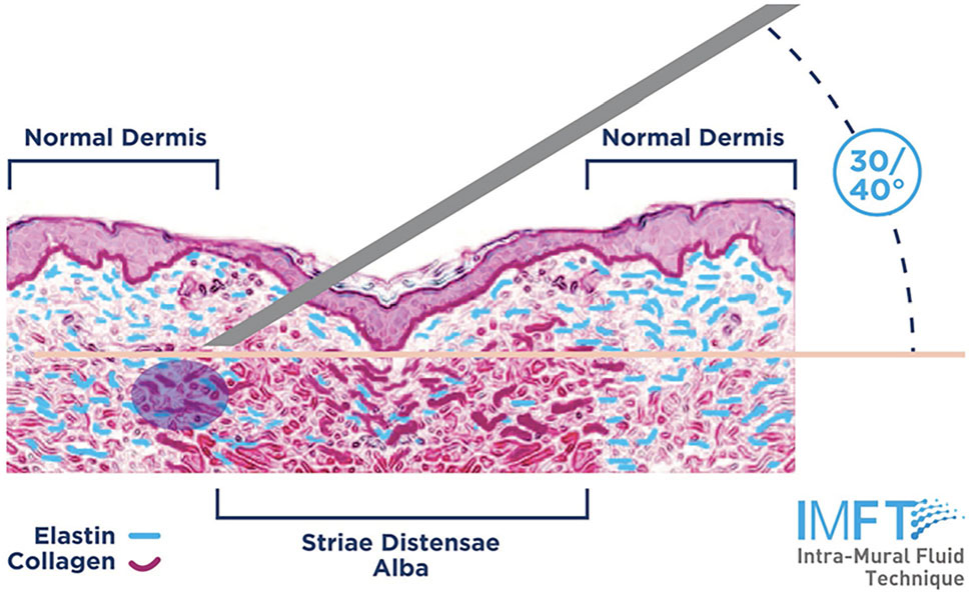
Intra-mural Fluid Technique (ImFT). The technique consists of the injection of 0.2–0.4 ml of the LMWHA + AA mixture using a 32–34 G needle. The inclination of the needle is 30/40° while the depth of penetration inside the wall of the SA was 3/4 mm

The treatment consisted of the injection of a mixture of a low molecular weight hyaluronic acid (LMWHA) and six amino acids (AA) (Sunekos Body®, Professional Dietetics, Milan, MI, Italy) in subjects with SA in one of the following areas: trochanteric, trochanteric/gluteal, thigh, periumbilical, or breasts. A maximum of 10 ml of LMWHA + AA were injected four times at 2 weeks interval each. The injection was performed using a 32–34 G needle, with an intra-mural fluid technique (ImFT), directly into the wall of every single SA with a needle inclination angle by 30/40° (Fig. 1), from superficial to deep dermis. Each stretchmark was treated in different points all over its length at about 3 mm each from the other. The volume of injection was 0.2–0.4 ml per point of injection.

LMWHA + AA is an injectable class III medical device composed of 10 ml of LMWHA and six AA (Glycine, L-Proline, L-Lysine, L-Leucine, L-Valine, L-Alanine) reconstituted in a specifically balanced solution adapting to the electrolyte composition of the interstitial fluids of the body (patent no. PCT/IB2015/059330).

### Outcome Measures

The improvement of the SA appearance was assessed by the investigator at T3 (after 15 days from the second treatment) and T5 (after 6 months and 4 treatments) using the Global Aesthetic Improvement Scale (GAIS) [32] (Table 1). GAIS scoring was based on the comparison of before and after images taken by the camera of an iPhone 13 smartphone (Apple Inc., Cupertino, California, US). The photograph scoring was performed using blinded analysis. The blinded analysis was performed by the same investigator evaluating unlabelled photographs presented in a random fashion at the end of the study. The improvement judgment was based on the decrease of the SA length and width as wells as on their overall appearance.

**Table 1:** GAIS scale

Score	Rating	Description
1	Very much improved	Optimal cosmetic result
2	Much improved	Marked improvement in appearance from the initial condition, but not completely optimal.
3	Improved	Obvious improvement in appearance from initial condition, but a re-treatment is indicated
4	No change	The appearance is essentially the same as the original condition
5	Worse	The appearance is worse than the original condition

**Fig. 2:**
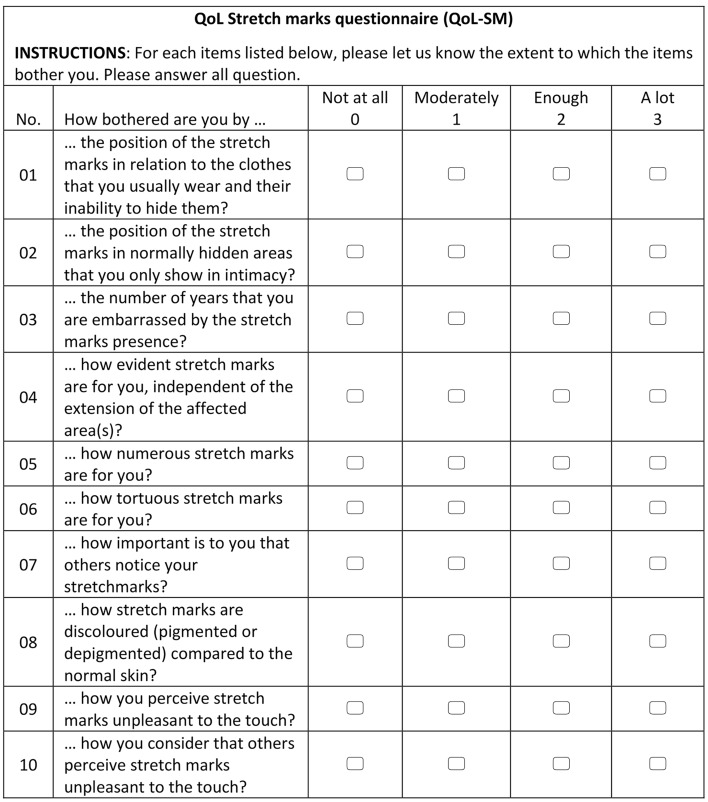
QoL-SM questionnaire

Likert Scale (from 1 = not tolerated at all to 5 = completely tolerated) was used to evaluate the tolerability after each treatment (T1, T2, T3, T4). The occurrence of side effect was scored by the investigator based on both physical (e.g., redness, swelling, desquamation, etc.) and functional signs (e.g., stinging, burning, etc.). The intensity of each sign was reported as follows: none (0), very mild (1), mild (2), moderate (3), severe (4). A quality-of-life stretch marks questionnaire (QoL-SM) was used to investigate the patients’ self-body image and self-esteem at T0 and T5. The QoL-SM consisted of a 10 items questionnaire and is a patient reported outcome to measure the appearance of stretch marks and their impact on the daily life. This QoL questionnaire asks the respondents to indicate how they are bothered by the appearance of the stretch marks in terms of both physical and emotional or psychosocial distress (Fig. 2). All these items were scored using the following scale: not at all (0), moderately (1), enough (2) and a lot (3). A decrease in the total score correlates with a decreased distress and an improvement of the initial condition.

### Statistics

The results reported in this paper are for the per protocol (PP) population and include all the subjects with complete data for all the endpoints.

All the data are reported as mean ± SE. A bilateral Wilcoxon signed ranked test was used for the statistical analysis of the QoL-SM data. The statistical analysis was carried out using NCSS 10 (version 10.0.7 for Windows; NCSS, Kaysville, UT, USA) running on Windows Server 2008 R2 Standard SP1 64-bit edition (Microsoft, WA, USA). A *p* < 0.05 was considered statistically significant.

## Results

Thirty-two (*n* = 32) female subjects were included in the study population. The per protocol (PP) population consisted of 29 subjects. The reason for not being included in the PP population were one of the following: withdrew due to personal reason (*n* = 2) and lost to follow-up (*n* = 1). The subjects were aged between 18 and 60 years old (38.0 ± 2.3 years old) with the following age distribution: 1 (3.4%) old adult aged ≥ 60 years old, 10 (34.5%) middle-age adults aged between 45 and 59 years old, 12 (41.4%) adults aged between 26 to 44 years old, and 6 (20.7%) young adults aged between 18 and 25 years old. The age distribution clearly indicates that the treatment was requested by subjects having an active social and relationship life, as can be expected.

The body weight at baseline was 56.2 ± 0.8 Kg and was unchanged during all the treatment period, as follows: 55.9 ± 0.8 Kg at T2, and 55.6 ± 0.8 Kg at T3/T4/T5. The stability of the body weight during the whole study duration was not then biasing the results.

**Fig. 3:**
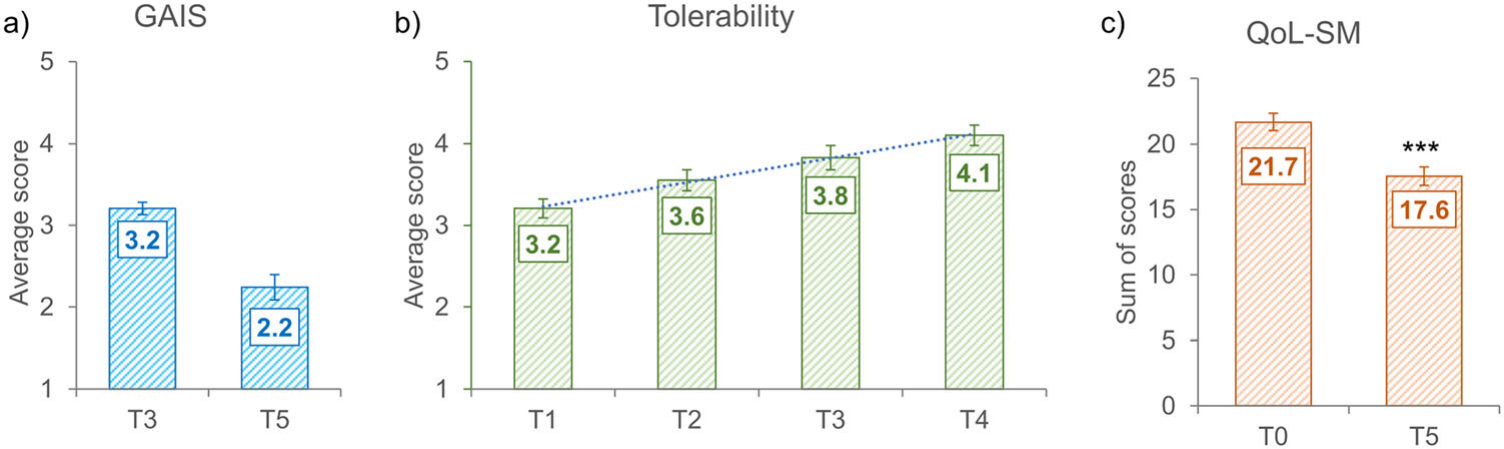
**a** Global esthetic improvement by GAIS scale. **b** Tolerability of the treatment by Likert scale. **c** QoL-SM questionnaire. Above the bars is reported the statistical analysis. Data are mean ± SE. *** *p* < 0.001.

**Fig. 4:**
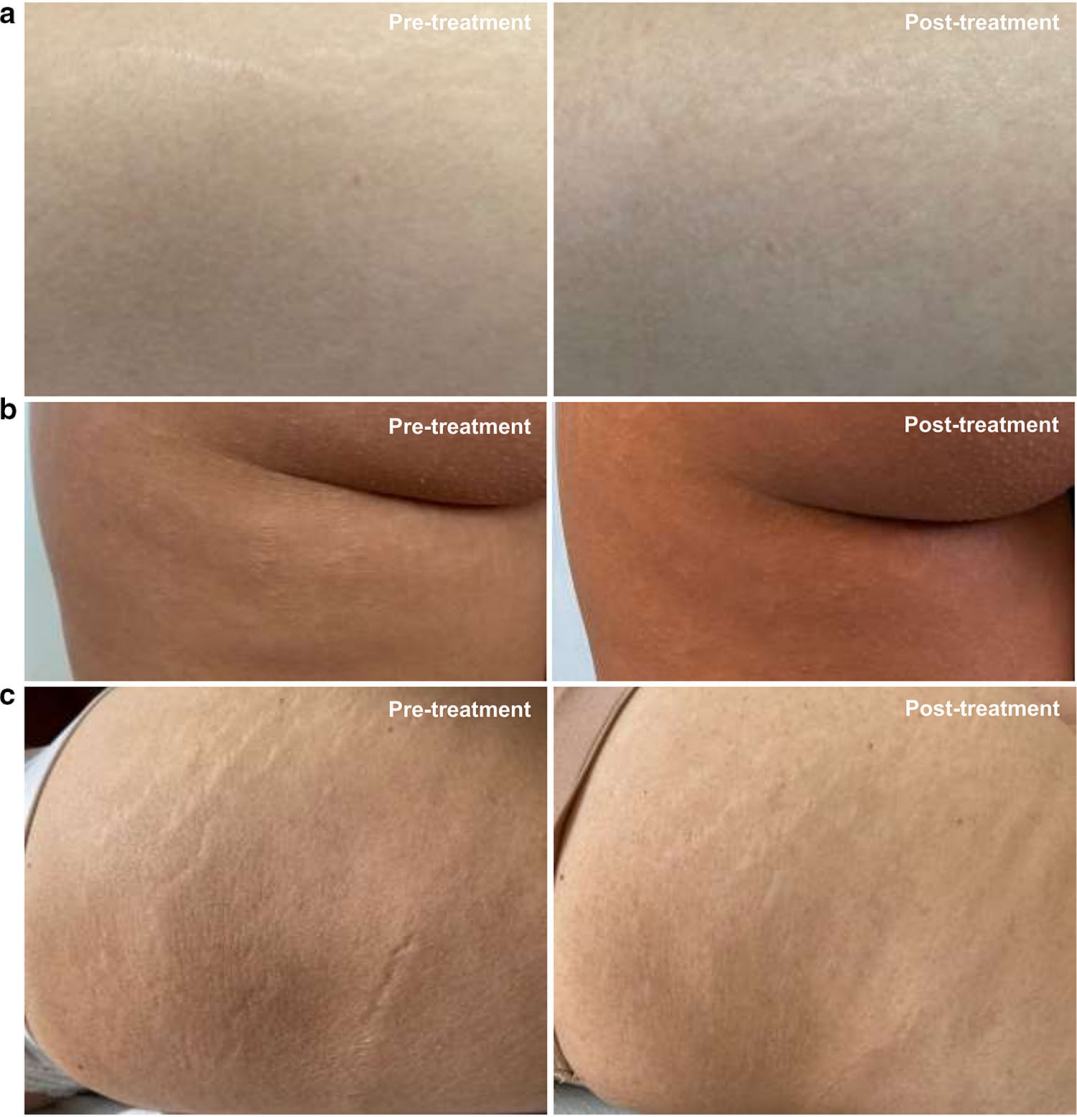
Improvement of clinical appearance of SA. **a** Subject 10 (21 years old) treatment of SA in the thigh area. **b** Subject 9 (19 years old) treatment of SA in the trochanteric-subgluteal area. **c** Subject 24 (60 years old) treatment of SA in the trochanteric-gluteus area

**Fig. 5:**
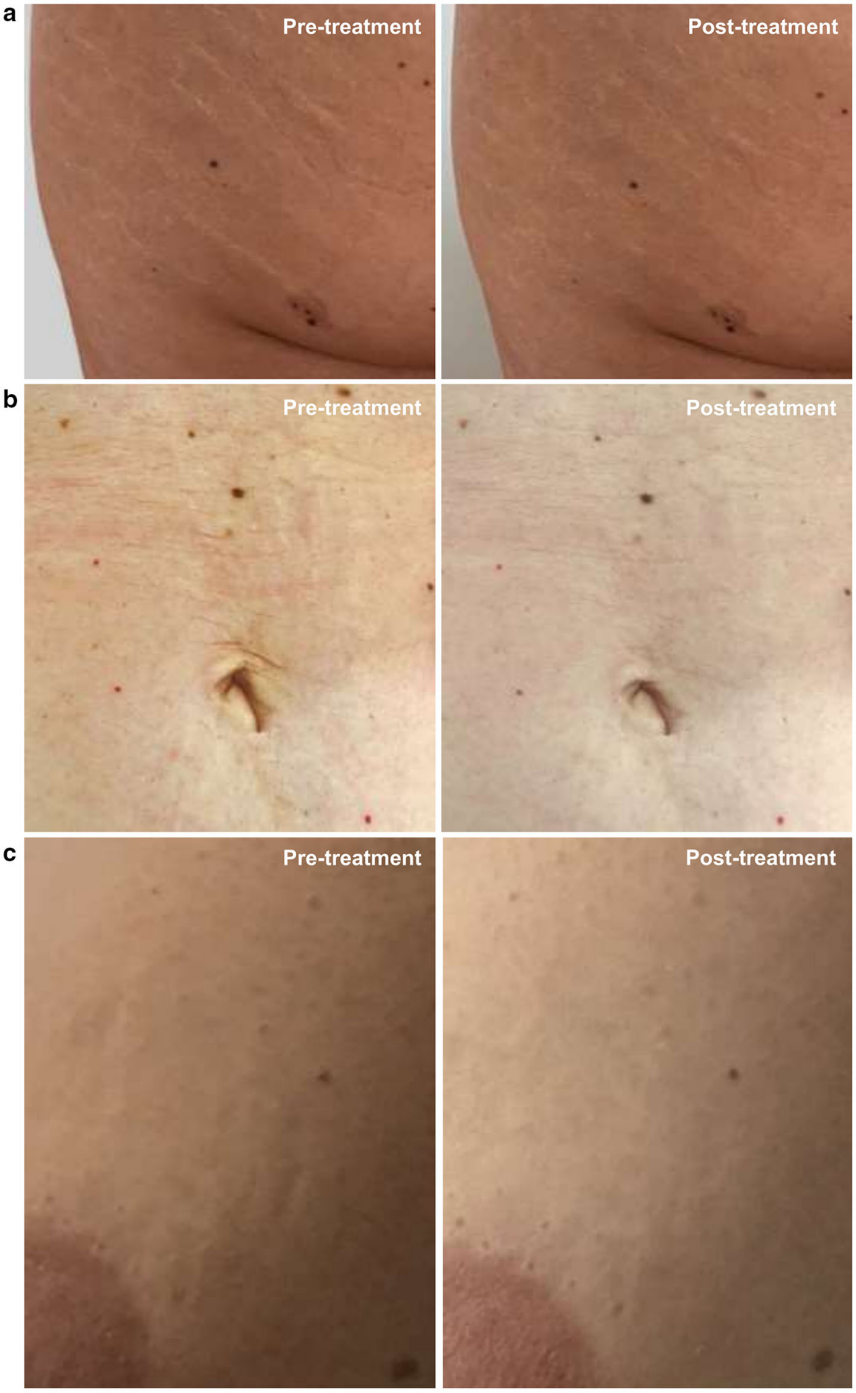
Improvement of clinical appearance of SA. **a** Subject 8 (41 years old) treatment of SD in the gluteal area. **b** Subject 11 (35 years old) treatment of SD in the abdomen area. **c** Subject 5 (26 years old) treatment of SD in the breast area

**Fig. 6:**
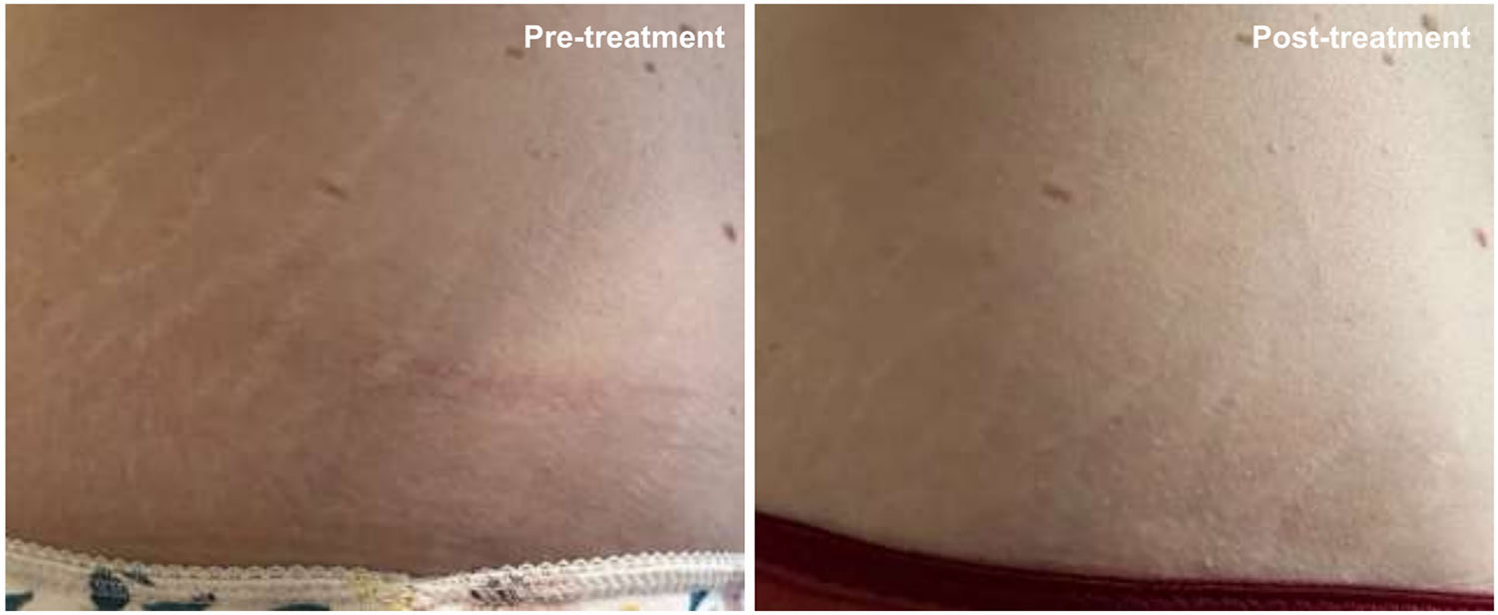
Improvement of clinical appearance of SA. Subject 25 (44 years old) treatment of SD in the hips area

Fifteen days after the second treatment (T3) the appearance of the SD, according to the GAIS scale, was improved (score = 3.2; rating: “improved”) (Fig. 3a), as follows: the 79.3% of the subjects were scored as “improved” while the 20.7% of them was scored as “much improved” (Table 2). Better results were obtained 6 months after the first treatment and after a total of 4 treatments, when the SA appearance was much improved (score = 2.2; rating: “much improved”), as follows: the 37.9% of the subjects were scored as “improved”, the 37.9% of the subjects were scored as “much improved”, and the 20.7% of the subjects were scored as “very much improved” (Figs. 4, 5, 6).

**Table 2:** Percentage distribution of GAIS and Likert tolerability scores

	T1	T2	T3	T4	T5
*GAIS*					
Score 1	…	…	0.0%	…	20.7% (↑)
Score 2	…	…	0.0%	…	37.9% (↑)
Score 3	…	…	79.3%	…	37.9% (↓)
Score 4	…	…	20.7%	…	3.4% (↓)
Score 5	…	…	0.0%	…	0.0% (=)
*Likert*					
Score 1	0.0%	0.0% (=)	0.0% (=)	0.0% (=)	…
Score 2	10.3%	6.9% (↓)	6.9% (↓)	3.4% (↓)	…
Score 3	58.6%	34.5% (↓)	20.7% (↓)	6.9% (↓)	…
Score 4	31.0%	55.2% (↑)	55.2% (↑)	65.5% (↑)	…
Score 5	0.0%	3.4% (↑)	17.2% (↑)	24.1% (↑)	…

… Assessment not foreseen at that checkpoint; = unchanged versus T1 (for Likert)/T3 (for GAIS); ↓ decreased versus T1 (for Likert)/T3 (for GAIS); ↑ increased versus T1 (for Likert)/T3 (for GAIS)

The treatment was tolerated by the subjects participating in the study with an improvement of the tolerability after each treatment (Fig. 3b), as follows: 3.2 after the first treatment (T1), 3.6 after the second treatment (15 days from the first treatment, T2), 3.8 after the third treatment (15 days from the second treatment, T3), and 4.1 after the fourth treatment (15 days from the third treatment, T4). The tolerability data confirmed the acceptability of this minimal invasive treatment.

The average baseline QoL-SM score was 21.7 ± 0.6 while its average distribution by age was as follows: 25.2 in the young adults (18–25 years old, *n* = 6), 22.3 in the adults (26–44 years old, *n* = 12) and 19.2 in the middle-age adults (45–59 years old, *n* = 10) population. The calculation was not performed for the old adults (≥ 60 years old) population, since it included only one subject. Interestingly, the QoL-SM score followed an age gradient in which the younger population was much more bothered by stretchmarks than the older population. Six months after the first treatment (and a total of 4 treatments) the patients’ QoL-SM score was statistically significantly (*p* < 0.001) improved (17.6 ± 0.7) by 19.4% (Fig. 3c). The improvement by age range was as follows: 18.8% (−9.5% min; −27.3% max) in the young adults, 17.1% (+4.2% min; −27.8% max) in the adults, 21.4% (−9.5% min; −27.3% max). The improvement for the old adults population was 31.6% but is related to only one subject.

## Discussion and Conclusions

The striae distensae, especially SA, are a challenging unesthetically dermal scarring condition, having an impact on the psychosocial wellness. A plethora of treatments have been proposed for SD including topical preparations, chemical peels, microdermabrasion, ablative and non-ablative lasers, micro-needling, dermal fillers, and others [32]. Despite the plethora of treatments available, none of them is advised as standard therapy [16, 33]. The laser therapy has been advocated as a treatment option for SD [6, 34, 35]. However, the laser therapy use is challenging in the clinical practice due to the high acquisition cost, maintenance, and the side effects of the procedure, including transient erythema, post-inflammatory hyperpigmentation, crusts, and pain [35].

In this study, we investigated the efficacy of a mixture of low molecular weight hyaluronic acid (LMWHA) and six amino acids (AA) when applied with a specific intradermal injection technique known as intra-mural fluid technique (ImFT) as a minimally invasive and effective treatment option for SA. In a previous study, Morganti et al. [36] reported the efficacy and safety of the injection of a mixture of hyaluronic acid sodium salt, sodium-carboxymethyl betaglucan and ascorbic acid on the SD area. Similar conclusion were drafted, recently, by Alsharif et al. [23] in their systematic review of the SD treatment with filler injection. The dispersion of HA injection was demonstrated to cause stretching of the skin surrounding the injection point and to active dermal fibroblasts [37–39]. The mechanical tension in the dermis triggers a series of reaction, including conversion of dermal fibroblasts to myofibroblasts by mechanoresponsive genes, acceleration of fibroblasts proliferation, and inhibition of apoptosis [40]. The healing process induced by the initiation of the natural inflammatory cascade could be also a putative mechanism beside the efficacy of the HA injection [41, 42].

Our study highlighted the safety and efficacy of the ImFT injection of LMWHA + AA. The treatment was tolerated by the patients. The tolerability on the Likert scale was improved over time, indicating an adaptation mechanism toward the “fear of needles” (trypanophobia) related to the injection procedure. No side effects were observed during the treatment except a mild stinging sensation during injection. At the end of the treatment period (T5), there was a marked improvement in the appearance of the SA when compared to the initial condition. This improvement was also perceived by the patients that reported an improvement of their self-confidence related to the decreased appearance of the SA and an improved their body perception.

In the younger population, was predominant at the baseline the embarrassment for having stretch marks in body areas that cannot be covered by clothes (e.g., hips) or in body areas they would show without any embarrassment to their partner (e.g., gluteus) or at the seaside (e.g., trochanteric area), while in the older population was predominant the components related to the number of years they were embarrassed for having stretch marks. The decrease in the QoL-SM score is then related, both in the younger and in the older patients, to a) an improvement of the patients baseline worries reported here above, b) to a decrease of how patients were bothered for their own and others perception of the stretch marks unpleasantness to the touch, and c) to a decrease of stretch marks tortuous appearance. Based on this observation, the improvement of the QoL-SM stretch marks questionnaire can be related to an improvement of the subject’s self-confidence and a better body perception both in the younger and in the older population.

Interestingly, the entity of the improvement of both the GAIS scoring and the QoL-SM stretch marks questionnaire was not related to the age of the subject, indicating that the product efficacy is not limited by the age of the SA.

Whereas other treatment options can be precluded to many potential patients due to cost and limited geographic availability [43], the HA injection is a valid treatment option with very mild side effects related to the injection procedure. The treatment tolerability was also confirmed by the results of our study in which HA was injected together with amino acids. The average tolerability was scored 3.2 for the first injection and 4.1 for the second injection, on the Likert scale, indicating a low injection pain intensity and a good tolerability. Results obtained in the present study support the efficacy of the injection of a LMWHA + AA mixture by ImFT in improving the SA appearance. However, the main study limitation is related to the study design. Future research may consider greater focus on enhanced study design, including larger, long-term split-body, or split-SD head-to-head randomized comparative trials with biopsy and molecular studies. In conclusion, it seems realistic to state that LMWHA + AA injection by ImFT can be a valid treatment option for SA convenient for both the practitioner and the patient due to the relatively low acquisition cost and the very mild side effects related to the injection procedure.
